# Loss of nucleosome assembly protein 1 affects growth and appressorium structure in blast fungus *Magnaporthe oryzae*

**DOI:** 10.17912/micropub.biology.000520

**Published:** 2022-01-24

**Authors:** Shweta Panchal, Kaustuv Sanyal

**Affiliations:** 1 Molecular Mycology Laboratory, Molecular Biology and Genetics Unit, Jawaharlal Nehru Centre for Advanced Scientific Research, Bangalore, Karnataka, India; 2 Bharat Chattoo Genome Research Centre, Department of Microbiology & Biotechnology Centre, Faculty of Science, The Maharaja Sayajirao University of Baroda, Gujarat, India

## Abstract

Evolutionarily conserved nucleosome assembly protein Nap1 is involved in multiple cellular processes in eukaryotes. In this study, we wanted to explore the role of Nap1 in the life cycle of rice blast fungus* Magnaporthe oryzae*. The null mutant of *M. oryzae NAP1 *is viable. However, deletion of *NAP1 *leads to defects in growth, appressorium morphology, and appressorium turgidity. In the future, plant infection studies can be undertaken to find if these defects lead to compromised virulence of this economically important fungal pathogen.

**Figure 1:**
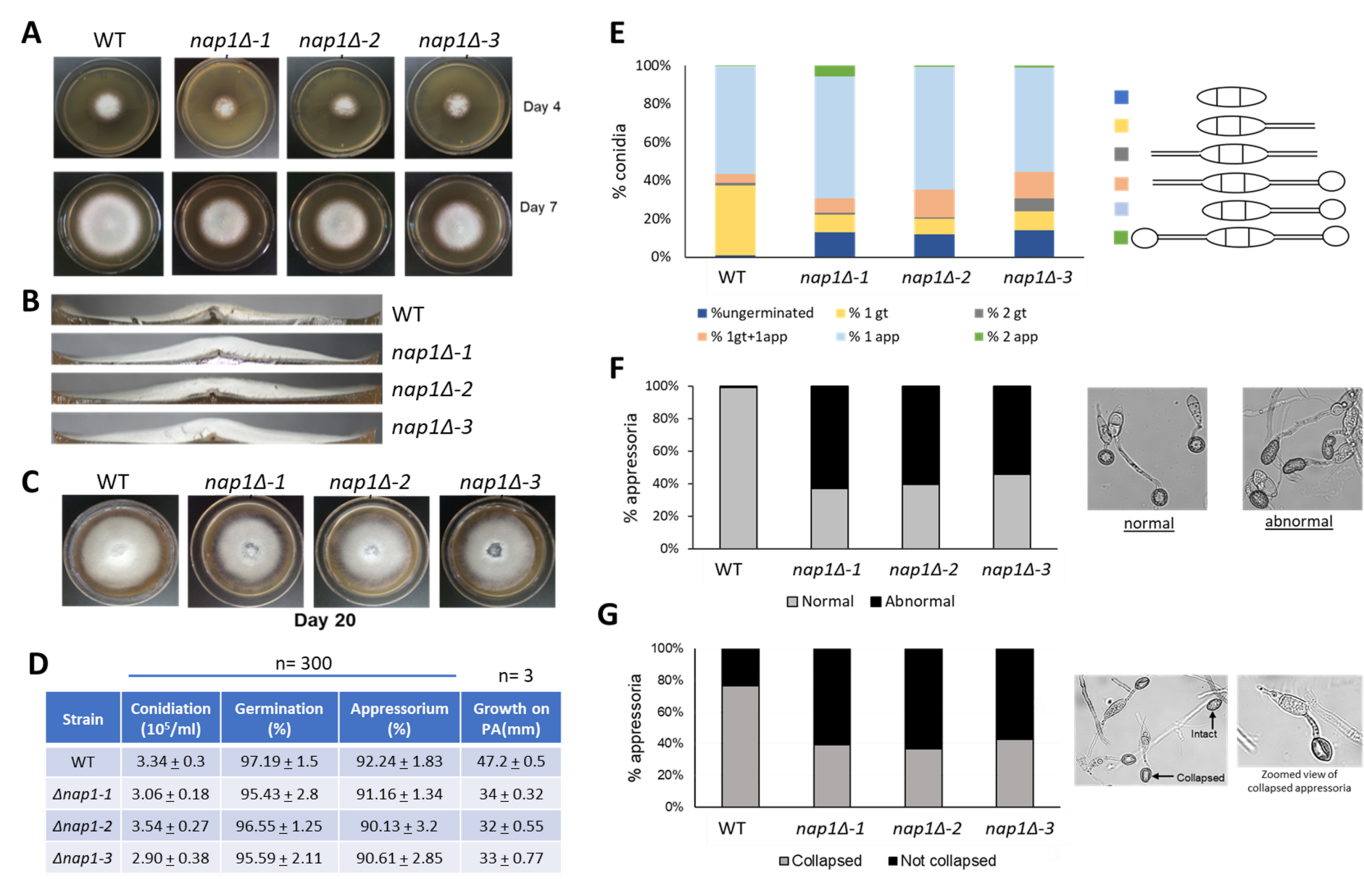
**Figure 1**Deletion of the *NAP1* gene leads to defects in colony morphology, appressorium formation, and appressorium turgidity. A, The typical colony size of wild-type and *nap1Δ* mutant strains on Day-4 and Day-7 post-inoculation. B, Transverse section of an 8-day old colony of indicated strains showing thickness of aerial hyphae. C, Early aging of mutant colonies as observed by autolysis in the middle of the plate. D, Quantification of phenotypic characteristics of wild-type and *NAP1* null mutants. E, Graph indicating the percentage of conidia that are either ungerminated, with one germ tube (1 gt), two germ tubes (2 gt), 2 germ tubes with one of them forming appressorium (1 gt+1 app), one germ tube forming one appressorium (1 app), or two germ tubes forming two appressoria (2 app) in an appressorium assay. n=100. F, Graph showing percentage of appressoria formed that are normally shaped versus abnormally shaped. n=>200. Representative micrographs showing normal versus abnormally shaped appressoria. G, Graph for the percentage of appressoria that collapsed on addition of 3M glycerol in the indicated strains. n=>500. Representative micrographs showing intact versus collapsed appressoria on addition of 3M glycerol.

## Description

Rice blast, caused by *Magnaporthe oryzae*, is a devastating plant disease with epidemics leading to losses of up to 30% of rice harvest yield (Wilson and Talbot 2009). This organism tops the list of ten major fungal pathogens in molecular plant pathology, owing to concerns over food security and economy (Dean *et al.* 2012). *M. oryzae* has a unique infection cycle. Infection is initiated when a fungal spore or a conidium attaches itself to the rice leaf cuticle and on receiving the suitable environmental cues, germinates to give rise to a single polarised germ tube. The germ tube later differentiates into a dome-shaped infection structure called the appressorium. Immense turgor pressure is generated within the appressorium which leads to mechanical disruption of the underlying leaf cell wall that facilitates the fungus to invade the plant cell where it proliferates and spreads in the plant tissue. In high humidity, the fungus sporulates out of the host and the spores are spread in the environment via rain splash and wind (Wilson and Talbot 2009). Elucidation of each of these steps is owed to results of extensive research in the last few decades which led to identification of several proteins and pathways involved in the infection cycle. This study was undertaken with the aim to understand the role of a core protein, nucleosome assembly protein (Nap1) in the life cycle of the blast fungus *M. oryzae*.

NAP proteins, as the name suggests, are involved in incorporation of histones during nucleosome assembly. They are highly conserved proteins present across all eukaryotes. Nap1 is involved in nucleosome remodelling, cell cycle regulation, transcriptional regulation, and shuttling of histones between the nucleus and cytoplasm (Zlatanova *et al.* 2007). *M. oryzae NAP1* (MGG_06024; *NAP1*) is a 1549 bp ORF with four introns. The spliced product (1215 bp) gives rise to a 404-amino acid polypeptide that has 46% identity with *Saccharomyces cerevisiae* Nap1. Using the gene replacement strategy, three independent *nap1Δ* mutants (*nap1Δ-1, nap1Δ-2, nap1Δ-3*) were generated and studied. Deletion of this gene was found to be nonessential and was observed to be associated with growth and appressorium formation*.* The *NAP1* null cells show defects in lateral spreading of the colony as observed by the smaller colony size (Fig. 1A). This may also indicate slower growth rate of these mutants. In addition, the mutant colonies appear fluffier with almost double the vertical size of the aerial hyphae mass than the wild-type (Fig. 1B). Colonies of *NAP1* null cells also underwent early senescence as seen by autolysis of the older cells at the middle of the colony in the plate (Fig. 1C), indicating that Nap1 may have a role in the aging process. The rate of sporulation in *nap1Δ* strains is similar to wild-type and *nap1Δ* conidia germinate and form appressoria within the same time as wild-type (Fig. 1D). However, defective germination was observed in a small percentage of mutant conidia. The number of conidia germinating with two germ tubes was more in the mutants, along with a few germ tubes with two appressoria (Fig. 1E). In addition, the appressorial morphology of mutants was distorted. While appressoria are usually circular or dome-shaped, about 50-60% appressoria in mutants were found to be elongated and abnormal (Fig. 1F). In yeast, Nap1 plays a role in regulating transition between polarized and isotropic growth (Kellogg and Murray 1995). While the polarized growth of the *M. oryzae nap1Δ* germ tube does transition to isotropic growth of the appressoria, it is not a normal transition. In the infection cycle of *M. oryzae,* the formation of the appressorium is highly critical for a successful infection. After the spore germinates to form an appressorium, solutes like glycerol and lipids accumulate and generate turgor pressure inside the appressorium. As much as 8 MPa of pressure is generated and with this sheer mechanical pressure, the appressorium breaches the plant cuticle and cell wall, and the fungus penetrates the host tissue (de Jong *et al.* 1997). To study if Nap1 is involved in generation of appressorial turgor, a cytorrhysis assay was performed (Galhano *et al.* 2017). An indirect estimate of appressorium turgor pressure is to measure the collapse of the appressorium on addition of exogenous glycerol. In this assay, the appressoria formed by *NAP1* null cells showed greater stability in the concentration of glycerol used, while 70-80% of wild-type appressoria collapsed (Fig.1G). This indicates that Nap1 may be involved in regulating appressorial turgidity. From the above results, we can conclude that Nap1 plays an important role in formation of mature dome-shaped appressoria with an optimum turgor pressure.

For future, it will be important to determine whether *nap1Δ* conidia (with abnormal appressoria) are able to successfully penetrate the leaf tissue and cause disease.

## Methods

- Strains and growth conditions:

*M. oryzae* wild-type (WT) B157 strain (MTCC accession no. 12236; Kachroo *et al.*, 1994) belonging to the international race IC9 was used in this study*. M. oryzae* B157 was grown on Prune agar (PA) or complete medium (CM) as described previously (Soundararajan *et al.*. 2004) at 28°C for 8-10 days. The colonies were cultivated on PA medium for 4 days in dark, followed by 4-5 days growth in constant illumination at room temperature. Vegetative growth was assessed by visual observation of the colony morphology and by measuring the colony diameter. Conidia were harvested as described previously (Patkar *et al.*, 2010), followed by microscopic observation of the conidial morphology. Harvested conidia were counted using a hemocytometer. For growth in liquid medium, small pieces of 8 day-old colony were used to inoculate in 30 mL CM at 28°C for 2-3 days. The biomass was collected by filtering through Miracloth (Calbiochem, Darmstadt, Germany) and drying using paper towels. The biomass was frozen and used for DNA extraction following standard protocols (Dellaporta *et al.*. 1983). Individual isolates were stored as filter stocks at -20°C.

- Gene replacement:

The sequence of Nap1 (MGG_06924) was obtained from *M. oryzae* genome assembly (Broad Institute, MIT). The knockout cassette was made by fusing three products – Nap1 upstream (Up), marker (Zeocin) ORF, and Nap1 downstream (Dn) in a molar ratio of 1:3:1 (Up:Zeo:Dn) by overlap PCR. The fused product was then amplified using XT-polymerase, precipitated and 2 µg of this amplified DNA was used for protoplast transformation of *M. oryzae*. Transformants were screened by negative PCR using Nap1 ORF primers and estimating band size differences using cassette primers and nested primers combinations.

- Protoplast transformation:

*M. oryzae* B157 was inoculated in 30 mL of CM broth and grown for 3 days. The biomass was then filtered with Miracloth, washed with sterile water and the mycelia were resuspended in 30 mL of 1M sorbitol containing 30 mg (1 mg/mL) *Trichoderma viridae* lytic enzymes (Sigma, St. Louis, USA). This was incubated at 28°C at 100 rpm for 12-16 hours for protoplasting. Next day, the protoplasts were filtered, washed and resuspended in 10 mL of 1 M sorbitol at 4000 rpm for 5 min at 4°C twice. The protoplasts were resuspended in 10 mL STC buffer (1 M sorbitol, 50 mM Tris-HCl pH 8, 50 mM CaCl_2_.2H_2_O), washed, and resuspended again in 1 mL STC buffer. The protoplasts were counted using a hemocytometer and a density of 10^8 ^cells/mL was maintained. For every transformation, 200 µL of protoplasts was mixed with 2-5 µg of DNA (dissolved in TE buffer) and incubated on ice for 15 min. To this 1 mL of PTC buffer (40% PEG 3350 + STC) was added and incubated at 28°C for 30 min. The whole mixture was transferred to a 15 mL tube containing 3 mL of YEGS (0.2% yeast extract, 1% dextrose, 1 M sorbitol) and was incubated at 28°C at 100 rpm for 12-14 hours. After incubation, 10 mL of molten regeneration medium (0.2% yeast extract, 1% dextrose, 0.4% agarose) was added, mixed well and poured on YEG agar containing 300 µg/mL Zeocin. Appearance of colonies was monitored within 2-3 days of incubation at 28°C. Water was used instead of DNA as negative control.

- Appressorium assay:

Fungal conidia were harvested by gently scraping off the biomass grown on PA plates using a sterile disposable plastic loop. The biomass was then filtered through two-layers of Miracloth. Spores were collected by centrifugation at 13,000 rpm for 4 min and re-suspended in water. Spore concentration of 10^5^ spores/mL was used and 10 µL of spore suspension was placed on hydrophobic cover-slip. This system was incubated in dark in a humid chamber for 12 hours, after which at least 100 conidia were imaged.

- Cytorrhysis assay:

Briefly, 10^5 ^spores were allowed to form appressoria for 18 h on coverslips after which water from the conidial sample was removed carefully by pipetting and replaced with 3M glycerol. After 10 minutes in glycerol, ~300 appressoria were imaged and analyzed.

All experiments were performed with three independent deletion mutants and at least twice with reproducible results.
